# Developing Ethical and Sustainable Global Health Educational Exchanges for Clinical Trainees: Implementation and Lessons Learned from the 30-Year Academic Model Providing Access to Healthcare (AMPATH) Partnership

**DOI:** 10.5334/aogh.2782

**Published:** 2020-10-26

**Authors:** Matthew Turissini, Tim Mercer, Jenny Baenziger, Lukoye Atwoli, Robert Einterz, Adrian Gardner, Debra Litzelman, Paul Ayuo

**Affiliations:** 1 Department of Medicine, Indiana University School of Medicine, Indianapolis, Indiana, US; 2 Department of Medicine, Moi University School of Medicine, Eldoret, KE; 3 Department of Population Health, The University of Texas at Austin Dell Medical School, Austin, Texas, US; 4 Department of Medicine, Indiana University School of Medicine and the Indiana University Center for Global Health, Indianapolis, Indiana, US; 5 Moi University College of Health Sciences Department of Mental Health, Aga Khan University Medical College East Africa, KE; 6 College of Health Sciences, Moi University, Eldoret, KE

## Abstract

**Background:**

There is strong interest among healthcare trainees and academic institutions in global health rotations. There are a number of guidelines detailing the ethical principles for equitable and ethical global health rotations and bilateral exchanges, but it is often challenging to know to implement those principles and develop longstanding partnerships.

**Objectives:**

The Academic Model Providing Access to Healthcare (AMPATH) is a 30-year continuous partnership between a consortium of 12 universities in North America and Moi University in Kenya. The AMPATH bilateral educational exchange has had 1,871 North American and over 400 Kenyan clinical trainees participate to date. The article describes the bilateral exchange of trainees including curriculum, housing, and costs and discusses how each is an application of the principles of ethical global engagement.

**Findings:**

The article takes the experiences of the AMPATH partnership and offers practical strategies for implementing similar partnerships based on previously published ethical principles.

**Conclusions:**

AMPATH provides a model for developing an institutional partnership for a bilateral educational exchange grounded in cultural humility, bidirectional relationships, and longitudinal, sustainable engagement.

## Introduction

Short Term Experiences in Global Health (STEGHs) are a popular and well-acknowledged valuable component of medical education. Thirty-one percent of United States (US) medical students graduating in 2015 had a global health experience during medical school, up from 15% in 1998 [1]. A growing number of trainees in U.S. residency programs are interested in global health as well, prompting an increase in global health education and international opportunities during post-graduate training [2,3,4,5]. However, there are significantly fewer opportunities for trainees from low- and middle-income countries (LMICs) to travel to clinical sites other than their own compared to the opportunities for trainees from North America [6,7,8,9,10]. Moreover, when providers from low- and middle-income countries do have clinical experiences in the US, they are typically limited to observation, unlike the frequent hands-on learning when the situation is reversed [38]. “Global health” can be both all-encompassing and vague. Consistent with the Alma Ata declaration, we use “global health” to mean *health for all, regardless of location or ethnicity* [39]. Global health may or may not include aspects of international medicine, tropical medicine, and public health, but must include a focus on the *wellbeing* of all aspects of the human experience (physical, social, environmental, spiritual) and be concerned with how health is achieved, with an emphasis on social determinants of health, health disparities, and transnational health solutions [40]. “Global health experiences” in our academic context indicate a dedicated focus on the health of a population different from one’s usual setting. For North American (NA) trainees, “global health” rotations, therefore, are typically in a low-income setting—either domestic or international. For trainees from low- and middle-income countries, “global health” electives indicate an experience either in a different low-income setting or in a higher-income setting such as NA or Europe.

## Ethical Principles for Bilateral Educational Exchanges

“Voluntourism” [41] and international experiences in which a medical trainee arranges a medical elective with little oversight or longevity are in stark contrast to the consensus of ethical principles for STEGHs and educational exchanges developed for academic institutions. Partnership with local institutions and the bilateral exchange of trainees are fundamental, critical aspects of equitable and ethically sound global health training experiences [10,11]. Guidelines for global health institutions and learners emphasize mutually beneficial relationships and grounding training experiences in bidirectional institutional commitment. The guidelines published by the Working Group on Ethics Guidelines for Global Health Training (WEIGHT) in 2010 encourage consideration of host needs and priorities, developing improvement and review processes, mentorship and supervision, and culturally-sensitive communication, among others [12]. Likewise, Melby et al. developed a set of institutional ethical principles with the goal of optimizing community benefit and learner experience for short-term experiences in global health [13]. These include: 1) cultural humility and building skills in cross-cultural effectiveness; 2) fostering bidirectional participatory relationships; 3) engaging longitudinally to promote sustainable local capacity and health system strengthening; and 4) embedding experiences within established, community-led efforts focused on sustainable development and measurable community health gains. Rowthorn et al. called for short-term experiences in global health to adhere to international laws and ethical standards [17,18], such as ensuring trainees operate within their scope of practice. Considerations of equality vs equity are paramount; for example, in medical education partnerships, institutions in LMICs compared to those in HICs often have fewer resources to support education, are in settings with fewer total health care providers, and have fewer faculty to provide care, teach, and directly supervise trainees [42,43,44]. Support for visiting trainees while on rotation so as not to burden faculty at the host institution is key.

These standards help the education community set a high bar for quality education in international settings with some programs striving to meet these guidelines [14,15,16]. However, despite established guidelines and these published examples, many STEGHs are not in line with these guidelines [19,20]. Moreover, many of these opportunities are not embedded within sustainable institutional partnerships and have potential for doing harm to host populations instead of the benefit they intend [20]. Despite good intentions, it can be difficult for institutions to know how to develop sustainable, ethical partnerships, and embed high-quality, ethical educational opportunities within them.

**Figure 1:**
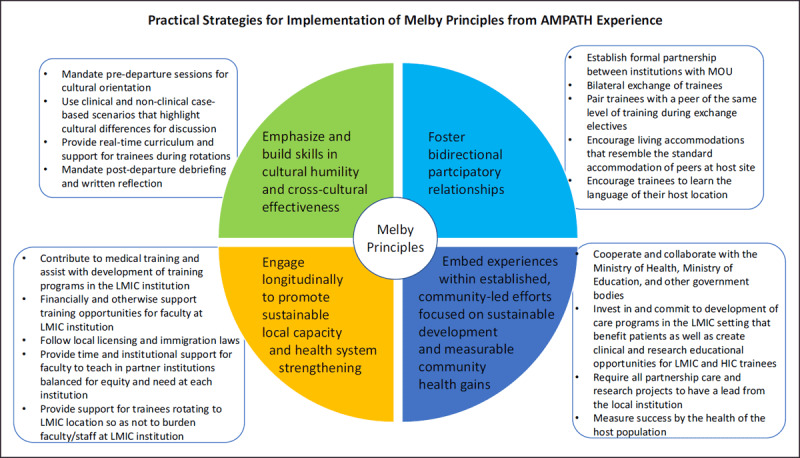
Practical Strategies for Implementation of Melby Principles from AMPATH Experience.

AMPATH, the Academic Model Providing Access to Healthcare, is a 30-year partnership between Moi University (MU), Moi Teaching and Referral Hospital (MTRH) in western Kenya, and a consortium of North American (NA) universities led by Indiana University (IU). In this article, we describe how AMPATH’s bilateral educational exchange of medical and pharmacy trainees has applied the ethical principles, particularly well summarized by Melby et al., for equitable and ethical global health educational partnerships and highlight some lessons learned (Figure 1). Importantly, we also outline how the bilateral educational exchange benefits from and augments the broader relationship of the trans-institutional partnership, which includes research and clinical care as well as education, and how the educational mission of the partnership supports both health system strengthening and local capacity building.

## AMPATH: The Process

### Getting Started

At its founding in 1990, Moi University sought international partners to help with curriculum development and medical education [21,22]. In support of this request for assistance, IU’s Division of General Internal Medicine committed to supporting a full-time faculty member at Moi University School of Medicine [23]. This initial investment helped seed the formation of a robust partnership between the two universities. Other NA universities (Table 1) joined the partnership beginning in 1997, ultimately forming the AMPATH Consortium (a name used to denote the NA partners collectively).

**Table 1:** Current AMPATH Member Institutions and Primary Areas of Involvement.

Member Institution	Primary Areas of Involvement

Moi University College of Health Sciences	Host Partner
Moi Teaching and Referral Hospital	Host Partner
Indiana University	Administrative leadership, Anesthesiology*, Dermatology*, ENT*, Hematology-Oncology*, Infectious Disease*, Informatics*, Internal Medicine*, Nephrology*, Nursing*, Palliative Care*, Pediatrics*, Public Health, Reproductive Health, Surgery*
Brown University	Biostatistics*, Dermatology, Family Medicine*, Gynecology-Oncology, Nephrology, Psychiatry*, Pulmonary
Purdue University	Agriculture*, Engineering*, Pharmacy*
Duke University	Cardiology*, Infectious Disease, Neurology, Nursing, Public Health, Pulmonary/Critical Care
University of Toronto	Adolescent Health, Public Health, Reproductive Health*
Icahn School of Medicine at Mount Sinai	Adolescent Health*, Palliative Care
University of Alberta	ENT, Plastic Surgery
University of California San Francisco	Dermatology, Radiology
University of Texas at Austin Dell Medical School	Neurology, Nursing, Pediatrics, Lead for AMPATH- Mexico replication site
Johns Hopkins University	Public Health, Monitoring and Evaluation
Stanford University	Family Medicine
New York University	Biostatistics, Cardiovascular Health, Radiology, Lead for AMPATH-Ghana replication site

All institutions participate across the trifold mission of clinical care, research, and the bilateral educational exchange. Participation and leadership in some fields is dynamic with significant overlap and collaboration. * Lead anchor institution. Anchoring departments lead efforts in conjunction with Kenyan partners.

### Building and Strengthening the Partnership

Over the three decades of educational exchange, involvement has expanded from a few NA and Kenyan faculty members to an inter-professional host of trainees and faculty from multiple institutions and disciplines including medicine, pharmacy, nursing, dentistry, public health, engineering, agriculture, law, business, and journalism. As a pre-condition for NA trainee rotations in Kenya, NA institutions must be members of AMPATH. Membership involves a commitment to a shared mission statement, hosting Kenyan trainees in bilateral exchange, adherence to AMPATH’s standard operating procedures for research and education, and faculty commitment to partnering with Kenyan counterparts within the care, education, and research programs. Most NA institutions lead in one or more specific clinical (e.g., cardiology) or cross-cutting (e.g., informatics) areas, although there is much crossover and collaboration across institutions and disciplines (Table 1). NA institutions pay annual dues to support the overall administrative and operational infrastructure of AMPATH and participate in regular forums for communication and exchange of ideas.

### Service

**Figure 2:**
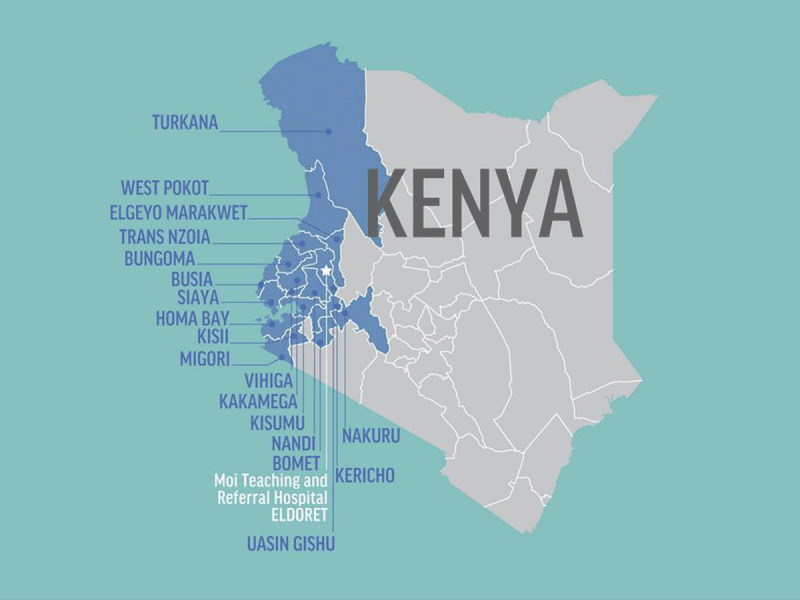
Map of Counties with AMPATH Care Programs.

AMPATH initially focused on community-based primary care within the health care delivery system of Kenya’s Ministry of Health. However, the emphasis shifted as the HIV epidemic consumed Kenya in the late 1990s [24,25]. Starting the first HIV treatment program in western Kenya, AMPATH grew exponentially and now provides services in over 500 Ministry of Health sites with over 160,000 people actively receiving HIV care complemented by comprehensive economic development, social support, and prevention programs (Figure 2) [26]. As the care of patients with HIV shifted to lifelong, chronic disease management, AMPATH re-focused on developing comprehensive primary care alongside robust sub-specialty care delivery. In partnership with the Kenyan MOH, AMPATH is developing a population health care delivery model that consists of a comprehensive and unified MOH care system from primary to tertiary levels of care, inclusive of an insurance package for the poor ensuring universal health coverage for the population [27,28,29].

### Research

As previously described [30,31], AMPATH has developed a strong collaborative research program, receiving over $146 million USD in extramural funding, producing over 680 peer-reviewed publications, and supporting a number of research training programs in epidemiology, biostatistics, implementation science, HIV/AIDS, and cardiovascular diseases [30,31]. This research has helped generate new knowledge, collect data, translate lessons learned into improvements in local and global care systems, and provide opportunities for both NA and Kenyan trainees to participate in mentored research projects. Additionally, the OpenMRS, one of sub-Saharan Africa’s first electronic medical record systems, was developed at AMPATH and is now in use in over 64 countries [32]. Discussing the steps toward replicating the AMPATH research model is outside the scope of this paper.

### Education

The clinical care systems and research infrastructure are foundational for educational opportunities for Kenyan and NA trainees. In addition to the bilateral exchange of trainees to be described in this paper, AMPATH has developed additional educational initiatives including the development of MU masters in medicine programs (equivalent to U.S. clinical residency programs), the development of MU sub-specialty medical fellowships, a pharmacy residency program, advanced practice clinical officer training programs, and many other healthcare workforce and research training initiatives in Kenya [33,34].

## Kenya to North America Educational Exchange

### Trainee Characteristics

Each year, Moi University College of Health Sciences (MUCHS) students and registrars (physicians receiving specialty training, similar to NA residents) complete rotations at one of the NA AMPATH institutions. Over 340 medical students, 14 dental students, and 50 registrars from MU have participated in the exchange since 1995.

### Rotation Structure and Learning Objectives

Medical students from MU rotate in various clinical departments at NA AMPATH institutions, including general internal medicine, pediatrics, gynecology, and subspecialty fields. The options available are driven by a combination of student interest as well as where there are pre-existing departmental relationships between the NA institution and MU. MU registrars in medicine, pediatrics, psychiatry, surgery, and OB/Gyn rotate at NA institutions as well. The curriculum for medical students and registrars includes participation on medical ward teams and didactics that exist for peers of the same training level at the host institution. The details of the logistics and curriculum for those rotating at IU have been described in depth previously [9]. The level of participation in patient care varies by AMPATH institution and is based on their local institutional policies for visiting trainees. For example, at Indiana University, Kenyan medical students are given the duties and privileges of a third-year medical student (including obtaining histories, doing physical exams, completing supervised procedures, and EMR access). However, at some other consortium partner institutions, trainees are observers—shadowing the clinical preceptor but not having direct patient contact.

### Supervision

Kenyan students work alongside their NA counterparts under the standard supervision at the academic institution—most often under a NA intern, resident, and faculty on their team. At several institutions, the chief resident is the Kenyan trainee’s primary contact for issues that may arise.

### Pre-Departure Training and Post-Rotation Debriefing

MU and NA faculty and administration in Kenya facilitate pre-departure curriculum for the Kenyans trainees rotating in NA; the curriculum includes logistics (passport and visa acquisition, vaccinations, travel arrangements, etc) and cultural orientation. Upon return to Kenya, MU student participants complete a reflection and presentation about their experience to facilitate transformative learning.

### Housing

Housing is arranged by each AMPATH institution and is most commonly a rented apartment or house close to the main clinical site, similar to the typical lodging of the local medical trainees.

### Costs

To help mitigate the economic inequities that exist between NA and MU trainees and to support bilateral exchange, the NA institutions cover the costs associated with travel and housing for MU students and nearly all registrars. NA institutions solicit philanthropic funds or use institutional funds for this purpose.

### Outcomes

Long-term follow-up data of MU participants indicate the majority of respondents considered the exchange the most influential component of their medical training. AMPATH’s focus on improving health systems helps create work environments that promote quality patient care, support future innovation, and enhance academic career development. While 52% of respondents stated the experience in a NA medical training institution seeded an interest in seeking additional experiences outside Kenya, 93% ultimately established their medical practice in Kenya, most commonly citing family ties and a commitment to their home community and country [37].

## North America to Kenya Educational Exchange

### Trainee Characteristics

There have been 1,871 medical and pharmacy students and residents from NA universities who have participated in the educational exchange with approximately 40–60 participating per year. The majority of NA students and residents doing educational electives are in Kenya for 1–3 months; a minimum duration of 2 months is encouraged and is the median duration. NA trainees visiting in non-healthcare disciplines such as engineering, law, and business follow separate curricula and are outside the scope of this paper.

### Rotation Structure and Learning Objectives

The backbone of the rotation is integration into daily rounds and patient care at a Kenyan tertiary care hospital (MTRH) working alongside MU trainees. This clinical role is combined with didactics at MU School of Medicine as well as supplemental lectures for visiting trainees to learn about the Kenyan health system, diseases common in Kenya, and differences in management across settings. The curriculum (Table 2) is guided by the following learning objectives:

- partner with Kenyan peers to advance individual and collective medical competency;
- develop skills in effective cross-cultural communication;
- understand the clinical presentation and management of common diseases in Kenya;
- improve proficiency in history and physical examination skills;
- understand the systems of medical care delivery, health research, and health education in Kenya;
- and learn skills that will facilitate the practice of compassionate, cost-effective medicine in home settings.

**Table 2:** Curriculum for North American Trainees in Kenya.

Curriculum Component	Facilitators	Description

Integration with Kenyan Trainees of Similar Training Level in Inpatient Clinical Teams at MTRH	Team leaders, MU/MTRH Faculty, Registrars, and Students	Partnering of North American trainees with Kenyan peers on teaching clinical teams to provide patient care
Moi University Registrar Morning Reports, Journal clubs, and other Didactics	MU/MTRH Departments	Visiting residents are invited to join weekly morning reports, journal clubs, and protocol reviews as planned for the Moi registrars
MUSOM Student Didactics	MU Departments	Visiting students are invited to join didactics as planned for the Moi students
AMPATH Morning Reports	Team leaders	Case-based morning reports led by NA trainees with faculty facilitation focused on delivering high quality care
Afternoon Global Health Talks	Team leaders, North American and Moi faculty	Talks on clinical topics including maternal and child health, malnutrition, chronic disease management, and infectious and tropical diseases as well as broader global public health topics including counterfeit medications, safe surgery 2020, and designing health systems
Fireside Chat	Team Leaders, North American faculty	Discussions around topics from a humanities and social science perspective on how politics, economics, social norms, cultural behaviors, and ethics intersect with health care locally and globally. Examples: death and dying, research ethics, bilateral exchanges, and cultural representations of illness
AMPATH Research Talks	AMPATH and Visiting Researchers	Talks on ongoing and past research projects to which visiting students and residents are invited
MTRH/AMPATH Sub-specialty Care Experiences	MTRH/AMPATH Sub-specialty Care Staff	Trainees round in the Cardiac Care Unit and rotate through specialty care clinics being driven by AMPATH efforts
AMPATH Clinic Visit	North American faculty	North American trainees are invited to spend a day at an AMPATH clinic seeing patients to better understand the outpatient HIV and chronic disease care system

### Supervision

NA students on clinical rotations at MTRH are supervised by Kenyan and NA faculty members working locally in Eldoret. NA students work alongside their MU student counterparts, primarily under the supervision of MU registrars (residents) and MU faculty who attend ward rounds and provide clinical and didactic medical education.

One NA faculty in each department (Internal Medicine, Pediatrics, Surgery, OB/Gyn, and Pharmacy) serves as a Team Leader living in Eldoret. Each team leader is funded by the NA institution department and serves as a guest faculty member within their respective MU department with clinical and educational duties (e.g. rounding, providing lectures to Kenyan trainees, participating in care programs). With the goal of minimizing the burden on Kenyans for hosting, teaching, supervising, and meeting other NA trainees’ needs, the Team Leaders also provide onsite supervision of NA trainees overseeing logistics and safety, teaching cultural sensitivity, helping trainees navigate a new health system, and guiding the educational curriculum. The Team Leaders serve one- to three-year terms in Kenya. These terms are intentionally longer than many other international positions to provide stability, develop expertise in teaching roles for Moi University trainees, facilitate involvement in long-term partnerships with Kenyan faculty in creating and supporting care, education, and research projects, and to serve as a professional development stepping stone to a career in global health. Team leaders work closely with Kenyan faculty clinically and in creating curriculum for NA and Kenyan trainees.

### Housing

Medical students, residents, researchers, and visiting faculty stay in a housing complex maintained by IU within walking distance to MTRH. Shared space for meals and lodging results in an environment conducive to adult learning with group discussions, critical reflection, and interdisciplinary collaboration [45,46]. Medical students stay a portion of their visit in the Kenyan medical student dormitory—a unique opportunity that is distinct from typical segregated models of housing that allows for a more immersive experience and facilitates the development of counterpart relationships that are the bedrock of the program.

### Costs

Most students and residents pay for their own travel and housing, though a few schools or departments have scholarship funds to support travel. Most residents continue to earn their normal salary while on the rotation, but this is subject to local institutional Graduate Medical Education policies.

### Pre-Departure Training and Post-Rotation Debriefing

NA AMPATH universities each have their own infrastructure for the preparation and debriefing of trainees. Each university provides pre-departure training in cultural awareness, program history and goals, and rotation expectations, as well as assistance with travel logistics such as vaccinations, visas, and licensing. A common online handbook developed by the IU Center for Global Health/AMPATH is provided to all visitors, and team leaders continue orientation after arrival in Kenya.

At the end of rotations, debriefing is done as individual or small group discussions with NA team leaders prior to departure from Kenya, and then with university global health staff after return home. Many NA institutions require a written reflection paper and/or oral presentation as well to help learners interpret, process, and grow from their experience [48,49].

### Outcomes

All trainees who complete the rotation are surveyed for qualitative and quantitative feedback via the university evaluation system, by email, and at the required debriefing. Long-term follow-up of participants from IU showed that compared to similar controls, AMPATH exchange participants were more likely to provide care to underserved populations, consider cost in clinical-care decisions, and be involved in public health policy and advocacy [35]. A qualitative study with 137 IU students and residents discussing their experiences in the bilateral exchange found four major themes: opening oneself to a broader world view, impact of suffering and death, life changing experiences, and commitment to care for the medically underserved. This analysis supports the effectiveness of the exchange for NA trainees as “a transformative learning experience that fosters the development of global mindedness and community involvement [36].”

## Limitations, Challenges, and Lessons Learned

The challenges of getting multiple academic institutions, each with their own priorities, strengths, barriers, and personalities, to send and receive students across an ocean are myriad. As one example, an area of effort currently is to understand the content and nature of the pre-departure training offered by each NA institution and to improve and standardize it.

The ratio of NA to Kenyan trainees participating in the exchange is weighted on the NA side. Cost is the primary explanation; philanthropic and departmental monies fund the costs of Kenyans completing electives in NA, while NA trainees typically pay for their own rotations. The AMPATH consortium balances the unequal numbers of trainees in the bilateral exchange through providing additional educators in Kenya, supporting Kenyan faculty development, providing equipment for Kenyan medical students, and assisting with creation of new training programs in Kenya.

Funding is a perpetual challenge in academic environments, and building support for global health programming is no exception. Creative funding methods have been required in addition to ongoing departmental support. Many programs across NA are developing global health fellowships, in which (an often junior) faculty member spends part of their time earning clinical dollars and part of their time abroad; this is attractive but may prohibit the long-term presence abroad that is fundamental for building strong connections and meaningful longitudinal contributions. The importance of building the connection between the team leaders who spent their time in Kenya and their home institutional department has proved important for the team leaders’ professional development.

Leadership within AMPATH is committed to a long-term partnership. The goal of the partnership is to strengthen both institutions, and the ideal is not to end the partnership once financial or educational support for the institution in the LMIC is no longer needed from partners in the HIC. Members of the NA AMPATH partner institutions have come to more fully appreciate the insight and understanding of global health challenges in our own backyards that are gained only through sustained partnership with our Kenyan partners. The shift in emphasis on cultural humility rather than cultural competency [50] is reflected in the trend toward reciprocal innovation—the idea that medical trainees, providers, and researchers can learn from each other no matter their country or culture of origin [47].

## Conclusion

The AMPATH educational exchange is dependent on the robust, longstanding partnership between the NA institutions and MU/MTRH, the commitment of NA institutions to have full-time faculty locally in Kenya, the commitment of NA universities to host and finance MU trainees rotating at their institutions, and MU departments welcoming of NA faculty into their departments and trainees on their wards. While these are seemingly large “asks” for university departments to make, AMPATH has demonstrated that this model is achievable and can lead to significant educational benefits for all involved.

Education partnerships would benefit from further study on the impact of bilateral rotations on trainees and the global workforce, the creation of best practices for pre-departure orientation and post-rotation debriefing, and policy change that supports trainees and faculty from LMICs rotating in NA [38]. Moreover, the global health community’s grand challenge for the future is to anchor additional institutional partnerships to create ethically sound and sustainable academic partnerships preparing a new generation of global health trainees to improve the health of populations and achieve health equity globally.
